# Performance evaluation of ML models for preoperative prediction of HER2-low BC based on CE-CBBCT radiomic features: A prospective study

**DOI:** 10.1097/MD.0000000000038513

**Published:** 2024-06-14

**Authors:** Xianfei Chen, Minghao Li, Xueli Liang, Danke Su

**Affiliations:** a Department of Medical Imaging Center, Guangxi Medical University Cancer Hospital, Nanning, Guangxi, China; b Department of Radiology, The First Affiliated Hospital of Hainan Medical University, Hainan Medical University, Haikou, China.

**Keywords:** breast cancer, contrast-enhanced cone-beam breast computed tomography, human epidermal growth factor receptor 2-low breast cancer, machine learning, radiomics

## Abstract

To explore the value of machine learning (ML) models based on contrast-enhanced cone-beam breast computed tomography (CE-CBBCT) radiomics features for the preoperative prediction of human epidermal growth factor receptor 2 (HER2)-low expression breast cancer (BC). Fifty-six patients with HER2-negative invasive BC who underwent preoperative CE-CBBCT were prospectively analyzed. Patients were randomly divided into training and validation cohorts at approximately 7:3. A total of 1046 quantitative radiomic features were extracted from CE-CBBCT images and normalized using *z*-scores. The Pearson correlation coefficient and recursive feature elimination were used to identify the optimal features. Six ML models were constructed based on the selected features: linear discriminant analysis (LDA), random forest (RF), support vector machine (SVM), logistic regression (LR), AdaBoost (AB), and decision tree (DT). To evaluate the performance of these models, receiver operating characteristic curves and area under the curve (AUC) were used. Seven features were selected as the optimal features for constructing the ML models. In the training cohort, the AUC values for SVM, LDA, RF, LR, AB, and DT were 0.984, 0.981, 1.000, 0.970, 1.000, and 1.000, respectively. In the validation cohort, the AUC values for the SVM, LDA, RF, LR, AB, and DT were 0.859, 0.880, 0.781, 0.880, 0.750, and 0.713, respectively. Among all ML models, the LDA and LR models demonstrated the best performance. The DeLong test showed that there were no significant differences among the receiver operating characteristic curves in all ML models in the training cohort (*P* > .05); however, in the validation cohort, the DeLong test showed that the differences between the AUCs of LDA and RF, AB, and DT were statistically significant (*P* = .037, .003, .046). The AUCs of LR and RF, AB, and DT were statistically significant (*P* = .023, .005, .030). Nevertheless, no statistically significant differences were observed when compared to the other ML models. ML models based on CE-CBBCT radiomics features achieved excellent performance in the preoperative prediction of HER2-low BC and could potentially serve as an effective tool to assist in precise and personalized targeted therapy.

## 1. Introduction

Breast cancer (BC) is a heterogeneous disease that varies among molecular subtypes. These subtypes differ in terms of frequency, prognosis, response to treatment, and survival outcomes.^[1]^ Approximately 45 to 55% of primary BCs exhibit low expression of the human epidermal growth factor receptor 2 (HER2), known as HER2-low.^[2–4]^ This categorization includes a wide range of cases, including triple-negative and luminal-type hormone receptor (HR)-positive breast tumors. These patients are classified as HER2-negative BC on the current HER2 validation guidelines, which means that HER2-targeted therapies are not recommended or effective in these patients.^[5]^ Trastuzumab deruxtecan, a novel antibody–drug conjugate, has shown promise in treating patients with HER2-low metastatic BC according to the results of the DESTINY-Breast04 study.^[6]^ According to recent clinical research, patients with HER2-low BC have unique clinicopathological traits, with varying prognoses and rates of pathological complete response compared to HER2-zero (immunohistochemistry [IHC] score 0) BC.^[7–9]^ Therefore, accurately distinguishing patients with HER2-low BC from HER2-negative BC is crucial for selecting the most appropriate therapy and predicting the treatment outcomes in this population.

In clinical practice, HER2 status can be determined based on IHC and/or in situ hybridization; however, owing to the heterogeneity of HER2 expression, approximately 15.3% and 7.3% of HER2-low BCs are mistakenly classified as HER2-negative and HER2-positive by core needle biopsy, respectively.^[10]^ Core needle biopsy is an invasive procedure. Thus, the development of noninvasive methods that can accurately and quickly determine HER2 status is imperative.

Contrast-enhanced cone-beam breast computed tomography (CE-CBBCT) is an innovative technology used for breast imaging that offers several advantages over existing modalities.^[11–13]^ It provides high spatial-resolution 3D images of the breast, allows for rapid data acquisition, and offers a comprehensive display of lesion morphology, calcification, and enhancement. CE-CBBCT has shown promising results in lesion identification and differentiation,^[14,15]^ molecular subtyping,^[16,17]^ assessment of disease extent,^[12,18]^ and prediction of axillary lymph node metastasis.^[19,20]^

Radiomics based on radiology medical images is a technique used in medical image analysis to uncover hidden factors within image pixels. This method involves manual or automatic collection of quantitative parameters from medical imaging, which may be associated with genotypic and molecular characteristics.^[21,22]^ The noninvasive nature of radiomics allows for the analysis and tracking of lesion distribution over time, eliminating the need for repeat biopsies. Machine learning (ML) algorithms play a crucial role in identifying and detecting significant radiomic features that support outcome predictions. By constructing predictive models using various ML algorithms and comparing their performance, the most effective predictive models can be extracted.^[23,24]^

Previous studies^[25,26]^ have demonstrated that ML models based on magnetic resonance imaging (MRI) radiomic features can predict HER2-low BC; however, whether CE-CBBCT radiomic features can discriminate HER2-low from HER2-negative BCs is uncertain. Therefore, this study aimed to develop and validate ML models based on CE-CBBCT radiomics features to predict HER2-low BC. In addition, we evaluated the effectiveness of various ML methods for determining the best prediction model.

## 2. Materials and methods

### 2.1. Study participants

**Figure 1.**
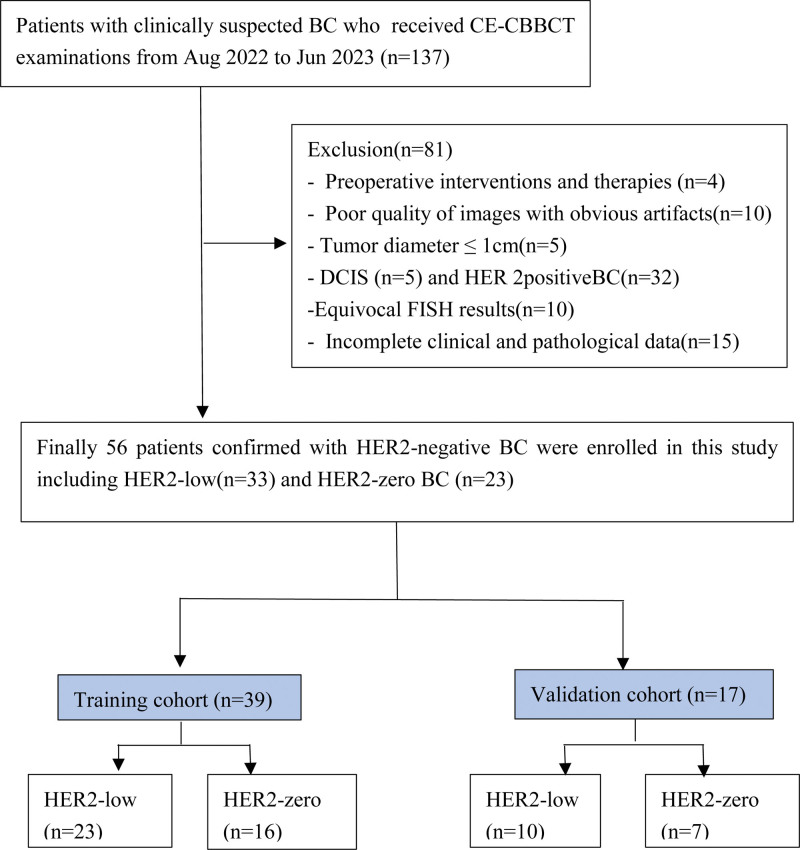
Patient enrollment flowchart with inclusion and exclusion criteria.

This prospective study was approved by the Institutional Ethics Board and each patient provided written informed consent. The inclusion criteria were as follows: patients with suspicious BC (detected by mammography or ultrasound) who underwent CE-CBBCT examinations at Guangxi Medical University Cancer Hospital (Nanning, China) between Aug 2022 and Jun 2023 and CE-CBBCT examinations completed 2 weeks before surgery.^[25]^ The exclusion criteria were preoperative interventions and therapies, poor image quality with noticeable artifacts, tumor diameter ≤ 1 cm, ductal carcinoma in situ and HER2 positive BC, equivocal FISH results, and incomplete clinical and pathological data. A total of 137 patients were enrolled in this study; 81 patients were excluded. Finally, 56 HER2-negative invasive BCs patients were enrolled, including 33 HER2-low and 23 HER2-zero cases. Patients were randomly assigned to the training and validation cohorts at a ratio of approximately 7:3. Figure 1 shows the patient flowchart of the study.

### 2.2. CBBCT protocol

The CBBCT system has been certified for breast imaging diagnostics by the National Food and Drug Administration of China and the USA specialized flat-panel detector breast CT scanner (Koning Breast CT, CBCT 1000, Koning Corporation, KBCT1000, Tianjin Corning Medical Equipment Co., Ltd.,Tianjin, PR China) was used for CBBCT in all the patients. The patients were positioned prone on the examination table and underwent breast scanning without compression. As previously reported,^[14,15,27]^ CE-CBBCT scans were performed with a constant tube voltage of 49 kVp^[27]^ and the tube current was automatically adjusted (between 50 and 160 mA) based on the size and density of the glandular tissues. A complete CE-CBBCT scan consists of 2 parts: non-contrast-enhanced and postcontrast-enhanced. Postcontrast scans were performed twice, 60 seconds (phase 1) and 110 seconds (phase 2) after the contrast medium was administered, as described in previous studies.^[14,15]^ For the CE-CBBCT scans, a nonionic iodinated contrast material (iohexol, 350 mg/mL; Yangtze River Pharmaceutical Co., Ltd., Jiangsu, PR China) was intravenously administered at a flow rate of 2 mL/s and a dose of 1.5 to 2.0 mL/kg with a dual-chamber power injector (MEDRAD Stellant D-CE, Bayer Medical Care Inc 1 Bayer Drive Indianola, PA 15051-07B0 USA), followed by a 30 mL bolus injection of saline solution at the same rate of 2.0 mL/s.

### 2.3. Image analysis

The imaging characteristics of the patients were evaluated by 2 radiologists with 15 (read 1) and 5 (read 2) years of expertise in breast imaging. During evaluation, both doctors were unaware of the patients’ pathological findings. The evaluation and reporting of CBBCT characteristics followed the recommendations specified in the American College of Radiology Breast Imaging Reporting and Data System atlas and lexicon, specifically the MRI and mammography sections. Two physicians arrived at a consensus regarding the interpretation of the following CBBCT features.

### 2.4. Clinicopathological data

To collect clinical and pathological data, a thorough evaluation of the patients’ medical records and pathological findings from the resected specimens was conducted. The expression of estrogen receptor, progesterone receptor, and HER2 was evaluated using avidin-biotin IHC following the guidelines established by the American Society of Clinical Oncology/College of American Pathologists.^[5]^ Positive estrogen receptor/progesterone receptor expression was defined as the presence of staining in >1% of tumor cell nuclei. A cutoff value of 20% was used to assess the Ki-67 index.^[25]^ HER2 status was confirmed postoperatively using IHC or FISH. Further FISH validation was performed for tumors with equivocal 2+ results. Based on FISH scores, patients were categorized into 4 groups: HER2-positive if a HER2 staining intensity score of 3+ or 2+ plus positive FISH was observed; HER2-negative if a score of 0, 1+, or 2+ plus negative FISH was obtained; HER2-low if a score of 1+ or 2+ plus negative FISH was obtained; and HER2-zero if a score of 0 was obtained.

### 2.5. Tumor segmentation

All DICOM format images from the first phase of CE-CBBCT were transferred to ITK-SNAP software (version 3.6.0; http://www.itksnap.org) for 3-dimensional (3D) segmentation of the region of interest (ROI). The radiologist (read 1) manually delineated the ROIs on all slices, ensuring accurate delineation of the tumor boundaries. To minimize the influence of the partial-volume effect, the selected ROIs were slightly smaller than those observed by the naked eye.^[28]^ To assess the stability of the features, 1 month later, both read 1 and read 2 independently performed segmentation on a randomly selected sample of 30 cases from the entire study set. The consistency and reproducibility of the features were evaluated using intraclass correlation coefficients (ICC). ICC values above 0.75 were considered indicative of good consistency.^[26,29]^

### 2.6. Radiomics feature extraction

Before extracting radiomics features, a series of image preprocessing steps were conducted, including gray-level discretization, intensity normalization, and voxel resampling to decrease feature variability.^[29–31]^ Wavelet imaging filters and Laplacian of Gaussian (LoG) filtering were used to process the original images and generate supplementary images to enhance the abundance of features. From the original, LoG, and wavelet images, 1046 quantitative radiomics features were extracted using PyRadiomics (https://github.com/Radiomics/pyradiomics).^[32]^ These included 18 first-order statistical features, 14 shape-based features (3D), 68 texture features, 688 wavelet features, and 258 LoG features. Radiomic features were extracted and standardized following the Imaging Biomarker Standardization Initiative.^[33]^ Detailed information on the formulae for calculating the radiomics features can be found at https://pyradiomics.readthedocs.io.

### 2.7. Feature selection and model building

To ensure reliable classification results and avoid any adverse impact on classifier performance due to sample imbalance, the synthetic minority oversampling technique^[34,35]^ was used to balance the datasets. Before reducing the dimensions, *Z*-score normalization was used to standardize the radiomic features and ensure a uniform scale of the feature values. To reduce the dimensionality of all radiomic features, Pearson correlation coefficient (PCC)^[26]^ was used to identify highly correlated feature pairs. If the absolute PCC was ≥0.99, the feature with superior performance was retained, indicating a strong correlation between the 2 features. Subsequently, the recursive feature elimination (RFE)^[35]^ method was used to obtain an optimal subset of features considering their redundancy and distinguishing power. The objective of RFE is to select features iteratively by reducing the feature set size at each iteration. Initially, the estimator is trained on the full set of features and the importance of each feature is determined using specific attributes (such as coef_ feature_importances_) or a callable function. Subsequently, the least important features were removed from the current set, and this process was repeated recursively on the pruned set until the desired number of features was achieved (more information can be found at https://scikit-learn.org/stable/modules/feature_selection.html#recursive-feature-elimination).

Six ML models, namely linear discriminant analysis (LDA), random forest (RF), support vector machine (SVM), logistic regression (LR), AdaBoost (AB), and decision tree (DT) (the details of the ML algorithms are described in the Supplementary Material, Supplemental Digital Content, http://links.lww.com/MD/M867 or can be found at https://scikit-learn.org/stable/user_guide.html#) were constructed using the optimal feature subsets selected by dimensionality reduction. An independent validation cohort was used to assess the predictive performance of the models, using the same thresholds as those determined in the training cohort. The performance was evaluated using a 10-fold cross-validation method, ensuring that the classes were roughly balanced in each fold.

**Figure 2.**
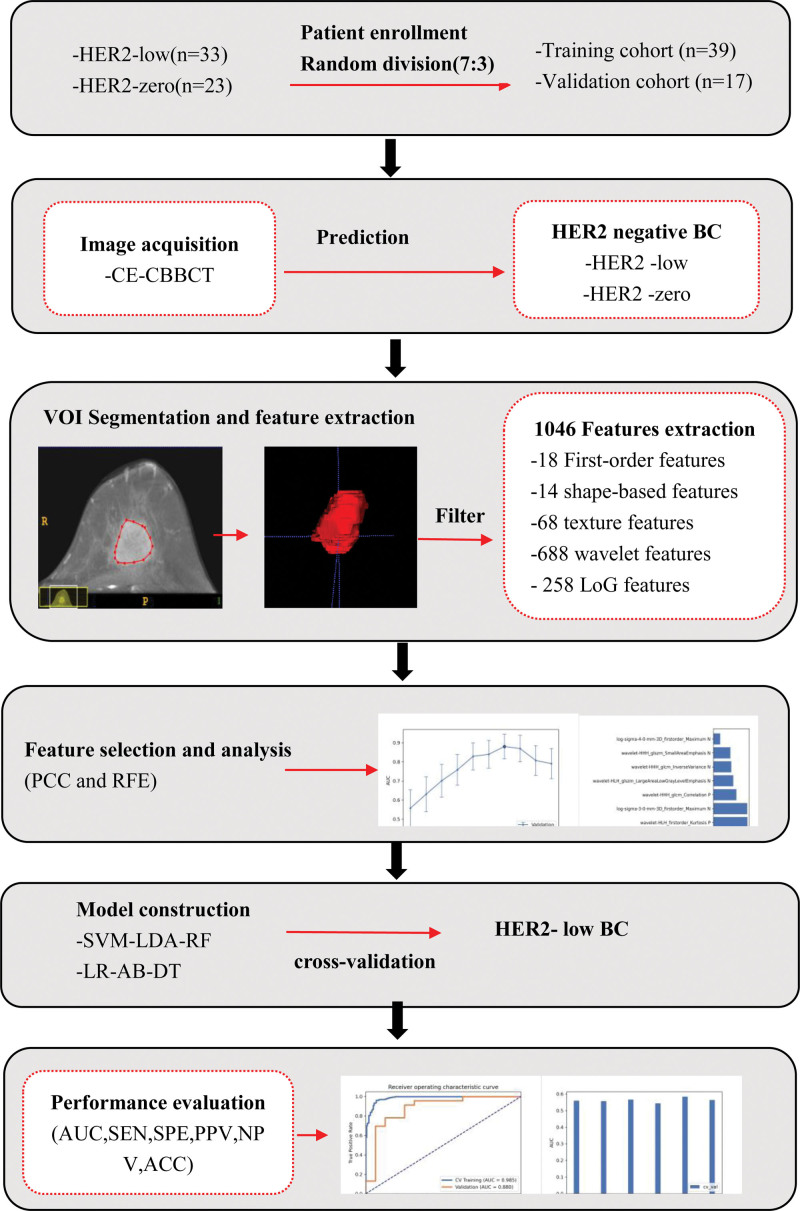
The radiomics workflow.

To evaluate the effectiveness of the aforementioned models, receiver operating characteristic (ROC) curves were drawn, and the area under the curve (AUC), accuracy, Matthews correlation coefficient, specificity, sensitivity, positive predictive value, and negative predictive value were calculated using FeAture Explorer Pro (FAE; v0.5.6) in Python v3.7.6. The FAE code can be found at https://github.com/salan668/FAE.^[36]^ Figure 2 shows the radiomics workflow.

### 2.8. Statistical analysis

The homogeneity and normality of the continuous variables were assessed using the Shapiro–Wilk and *F* tests. Continuous variables with normal distributions are expressed as mean ± SD, whereas skewed distributed variables are presented as medians (lower quartile, upper quartile). The classification data are expressed as the relative distribution frequency and percentage. Independent-sample *T*-tests and Mann–Whitney *U* tests were used to compare normal and skewed distributions, respectively. Categorical variables were compared using the chi-square and Fisher exact tests. The Delong test was used to compare the ROC curves between the models. All statistical analyses were 2-sided, and *P* < .05 was considered statistically significant. All statistical analyses were performed using SPSS 26.0 (IBM Corp., Armonk).

## 3. Results

### 3.1. Clinicopathological and CE-CBBCT characteristics

All patients were women with a mean age of 49.71 ± 10.45 years (range: 31–80 years). The patients were divided into training (n = 39) and validation (n = 17) cohorts. In the training cohort, 23 (59.0%) patients were HER2-low and 16 (41.0%) were HER2-zero; in the validation cohort, 10 (58.8%) were HER2-low and 7 (41.2%) were HER2-zero. The clinicopathological characteristics of the 2 cohorts were compared, and no significant differences were found (*P* > .05), as shown in (Table 1). Similarly, no significant differences in the CBBCT findings were observed between the training and validation cohorts (Table 2).

**Table 1:** Patient characteristics in the training and validation cohort.

Clinicopathological features	Training cohort (n = 39)	Validation cohort (n = 17)	*P*
Age (yr)	48.95 ± 10.06	51.47 ± 11.41	.411
Diameter (cm)	2.60 (1.90, 3.40)	2.80 (2.15, 4.00)	.368
Menstrual status, n (%)			.432
Premenopausal	25 (64.1%)	9 (52.9%)	
Postmenopausal	14 (35.9%)	8 (47.1%)	
ER, n (%)			1.000
Positive	33 (84.6%)	15 (88.2%)	
Negative	6 (15.4%)	2 (11.8%)	
PR, n (%)			.119
Positive	30 (76.9%)	16 (94.1%)	
Negative	9 (23.1%)	1 (5.9%)	
Ki-67, n (%)			.738
≤20%	9 (23.1%)	3 (17.6%)	
>20%	30 (76.9%)	14 (82.4%)	
Histological grade, n (%)			.533
I or II	28 (71.8%)	10 (62.5%)	
III	11 (28.2%)	6 (37.5%)	
ALN status (%)			.867
Positive	22 (56.4%)	10 (58.8%)	
Negative	17 (43.6%)	7 (41.2%)	
HER2-negative BC			.992
HER2-low	23 (59.0%)	10 (58.8%)	
HER2-zero	16 (41.0%)	7 (41.2%)	

Data are presented as mean ± SD, median (lower quartile, upper quartile), or number (%).

ALN = axillary lymph node, BC = breast cancer, ER = estrogen receptor, HER2 = human epidermal growth factor receptor 2, PR = progesterone receptor.

**Table 2:** CBBCT findings of patients in the training and validation cohorts.

CBBCT findings	Training cohort	Validation cohort	*P*
Breast density, n (%)			.242
a/b	12 (30.8%)	8 (47.1%)	
c/d	27 (69.2%)	9 (52.9%)	
No. of lesions, n (%)			.468
Single	31 (79.5%)	12 (70.6%)	
Multiple	8 (20.5%)	5 (29.4%)	
Tumor margins, n (%)			.527
Non-spiculated	29 (74.4%)	11 (64.7%)	
Spiculated	10 (25.6%)	6 (35.3%)	
Internal enhancement, n (%)			1.000
Homogeneous	4 (10.3%)	2 (10.5%)	
Heterogeneous	31 (79.5%)	16 (84.2%)	
Rim enhancement	4 (10.3%)	1 (5.3%)	
CBBCT-associated NME, n (%)			.151
Present	5 (12.8%)	6 (31.6%)	
Absent	34 (87.2%)	13 (68.4%)	
Suspicious calcification, n (%)			.394
Present	12 (30.8%)	8 (42.1%)	
Absent	27 (69.2%)	11 (52.9%)	
Vascular abnormalities, n (%)			.115
Present	29 (74.4%)	9 (52.9%)	
Absent	10 (25.6%)	8 (47.1%)	

CBBCT = cone-beam breast computed tomography, NME = non-mass enhancement.

### 3.2. Intra-observer and inter-observer agreement for radiomics features extraction

The intra-observer ICC ranged from 0.773 to 0.983, and the inter-observer ICC ranged from 0.787 to 0.980 for the evaluation of radiomics feature extraction. The results showed good consistency in feature extraction within and between observers (Table 3).

**Table 3:** Intra- and inter-correlation coefficients for optimal selected features.

Features	ICC (inter-)	ICC (intra-)
log-sigma-3-0-mm-3D_firstorder_Maximum	0.958	0.838
log-sigma-4-0-mm-3D_firstorder_Maximum	0.912	0.790
wavelet-HHH_glcm_Correlation	0.816	0.925
wavelet-HHH_glcm_InverseVariance	0.922	0.983
wavelet-HHH_glszm_SmallAreaEmphasis	0.787	0.842
wavelet-HLH_firstorder_Kurtosis	0.810	0.972
wavelet-HLH_glszm_LargeAreaLowGrayLevelEmphasis	0.980	0.773

ICC = intraclass correlation coefficients.

log-sigma-3-0-mm-3D_firstorder_Maximum

log-sigma-4-0-mm-3D_firstorder_Maximum

wavelet-HHH_glcm_Correlation

wavelet-HHH_glcm_InverseVariance

wavelet-HHH_glszm_SmallAreaEmphasis

wavelet-HLH_firstorder_Kurtosis

wavelet-HLH_glszm_LargeAreaLowGrayLevelEmphasis.

### 3.3. Prediction performance of ML models

**Figure 3.**
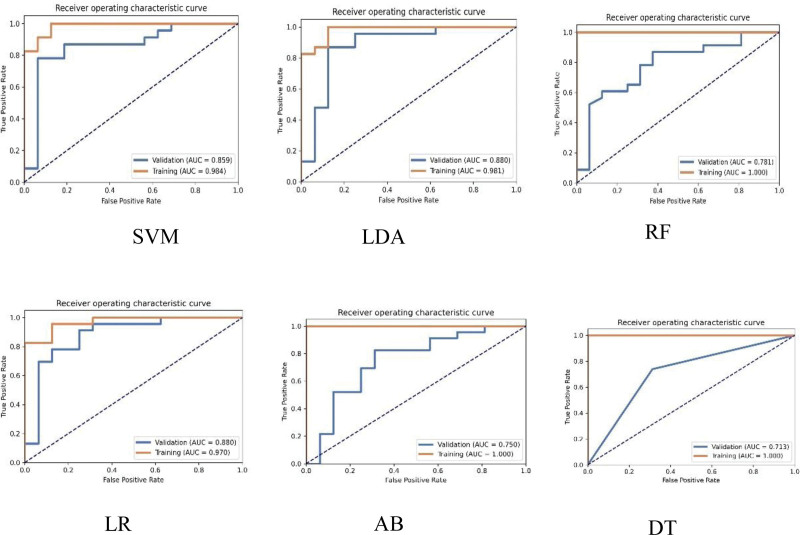
Receiver operating characteristic curves of machine learning model for predicting HER2 low breast cancer in the training and validation cohort. HER2 = human epidermal growth factor receptor 2.

As shown in Table 4, in the training cohort, the AUC of the SVM, LDA, RF, LR, AB, and DT were 0.984, 0.981, 1.000, 0.970, 1.000, and 1.000, respectively. In the validation cohort, the AUC of the SVM, LDA, RF, LR, AB, and DT were 0.859, 0.880, 0.781, 0.880, 0.750, and 0.713, respectively. Figure 3 shows the ROC curves of the 6 ML models. The DeLong test showed that there were no significant differences among the ROCs of all ML models in the training cohort (*P* > .05); However, in the validation cohort, the results of the DeLong test showed that the difference between the AUC of LDA and RF, AB, and DT was statistically significant (*P* = .037, .003, .046), while the DeLong test showed that the difference between the AUC of LR and RF, AB, and DT was statistically significant (*P* = .023, .005, .030). However, when compared with other ML models, there was no statistically significant difference. The results are summarized in Table 5.

**Table 4:** Evaluation indicators of the predictive performance of 6 models for predicting HER2-low.

Classifier		AUC	MCC	Acc	Sen	Spe	PPV	NPV
SVM	Training dataset	0.984	0.897	0.949	1.000	0.875	0.920	1.000
	Validation dataset	0.859	0.709	0.846	0.783	0.938	0.947	0.750
LDA	Training dataset	0.981	0.897	0.949	1.000	0.875	0.920	1.000
	Validation dataset	0.880	0.739	0.872	0.870	0.875	0.910	0.824
RF	Training dataset	1.000	1.000	1.000	1.000	1.000	1.000	1.000
	Validation dataset	0.781	0.516	0.769	0.870	0.625	0.769	0.769
LR	Training dataset	0.970	0.841	0.923	0.957	0.875	0.917	0.933
	Validation dataset	0.880	0.680	0.846	0.913	0.750	0.840	0.857
AB	Training dataset	1.000	1.000	1.000	1.000	1.000	1.000	1.000
	Validation dataset	0.750	0.519	0.769	0.826	0.688	0.792	0.733
DT	Training dataset	1.000	1.000	1.000	1.000	1.000	1.000	1.000
	Validation dataset	0.713	0.423	0.718	0.739	0.688	0.773	0.647

AB = AdaBoost, Acc = accuracy, AUC = area under the curve, DT = decision tree, HER2 = human epidermal growth factor receptor 2, LDA = linear discriminant analysis, LR = logistic regression, MCC = Matthews correlation coefficient, NPV = negative predictive value, PPV = positive predictive value, RF = random forest, Sen = sensitivity, Spe = specificity, SVM = support vector machine.

**Table 5:** DeLong test results for each receiver operating characteristic curve for the validation cohort.

Comparison	*Z*	*P*
SVM - LDA	−.563	.573
SVM - RF	1.364	.172
SVM - LR	−.817	.414
SVM - AB	1.737	.082
SVM - DT	1.749	.080
LDA - RF	2.087	.037
LDA - LR	.000	1.000
LDA - AB	2.978	.003
LDA - DT	1.993	.046
RF - LR	−2.281	.023
RF - AB	.845	.398
RF - DT	.955	.339
LR - AB	2.791	.005
LR - DT	2.175	.030
AB - DT	.407	.684

AB = AdaBoost, DT = decision tree, LDA = linear discriminant analysis, LR = logistic regression, RF = random forest, SVM = support vector machine.

## 4. Discussion

Our study demonstrated that certain radiomic features derived from CE-CBBCT can be used to predict HER2-low BC. The LDA and LR classifiers achieved the highest AUC (0.880) and demonstrated a superior performance in the validation cohort. However, the LDA classifier exhibited higher accuracy (0.872) than the LR classifier (0.846). In contrast, the DT model showed a lower AUC value (0.713), indicating a tendency toward overfitting. ML models based on CE-CBBCT radiomics have the potential to effectively predict HER2-low BC in a noninvasive manner. These models could be valuable for supporting clinical diagnoses and guiding treatment decisions.

To the best of our knowledge, this is the first study to investigate the correlation between radiomic features extracted from CE-CBBCT images and the HER2-low BC status. Bian et al^[25]^ conducted a study in which they utilized intratumoral and peritumoral radiomic characteristics from multiparametric MRI to differentiate HER2-low from HER2-zero breast tumors, achieving AUC of 0.776 and 0.71 in internal and external validation. Similarly, another study^[26]^ found that radiomic features extracted from multiparametric MRI were capable of differentiating HER2- low from HER2-zero BCs, achieving an AUC of 0.79. However, our study surpassed these previous findings in terms of superior predictive performance, achieving an AUC of 0.880. This disparity may be attributed to the fact that CE-CBBCT can simultaneously exhibit abnormal enhancement and calcifications, which is a remarkable advantage that MRI cannot achieve.^[12]^

Compared to HER2-zero, HER2-low BCs show a higher incidence of HR positivity and mutations in the PI3K/AKT/mTOR pathway, which have been identified as the main factors contributing to trastuzumab resistance.^[7,9]^ The clinicopathological and molecular characteristics of these 2 subtypes can be observed macroscopically in CE-CBBCT, whereas subtle variations can be quantified using high-throughput radiomics features. The HR status and PI3K/AKT/mTOR pathway may form the biological basis of this radiomic model. However, this hypothesis should be confirmed by future radiogenomic studies.

The generalizability of ML models benefits from feature selection, which aims to extract the most informative features while eliminating noisy, irrelevant, and redundant ones.^[37]^ The 3 main methods for feature selection are filtering, wrappers, and embedding methods.^[38]^ In this study, we employed PCC^[39]^ and RFE^[37]^ for feature selection and reduction. PCC is a widely used filter method that ranks features based on their correlation with a class, removing those below a certain threshold. Although less computationally intensive, filter methods may lead to models with reduced predictive performance compared to wrapper or embedded methods. RFE is^[37]^ a wrapper method that identifies features with minimal impact on performance when removed. Unlike filter methods, wrapper methods utilize the classifier’s performance as a metric for selecting the best feature subset, resulting in higher predictive performance. However, wrapper methods are influenced by the choice of the classifier.

In our study, ML models were developed by incorporating 7 optimal radiomic features including 2 LoG and 5 wavelet features. Both wavelet and LoG features highlight variations in gray-level regions within the tumor, capturing tumor heterogeneity more effectively than the simpler texture features. This can be attributed to the fact that higher-order filtering obtained through image transformation enhances the visibility of the subtle details of the tumor texture. Although the exact biological characteristics captured by these 2 features cannot be determined, it is hypothesized that these subtle details may be associated with slight changes in microvessel density and the tumor microenvironment.^[20]^

The current standardized techniques for evaluating HER2 expression are IHC and FISH, although disagreement has been reported between the central and local assessments of HER2-zero and HER2-low scores.^[40]^ Typically, this assessment is based on a single biopsy of a potentially heterogeneous tumor, providing only a local snapshot of its biological characteristics.^[35]^ By incorporating radiomic signatures as diagnostic tools into their workflow, pathologists can enhance their awareness of potential HER2 expression in tumors. Therefore, the radiomic signature could serve as a noninvasive biomarker for evaluating whole-tumor heterogeneity and assist in identifying the most appropriate target for biopsy to maximize the possibility of detecting HER2 expression in cases of multifocal involvement. Furthermore, HER2 expression can be repeatedly assessed to monitor changes in the spatiotemporal biology of tumors as the disease progresses. Considering the limited treatment options available for resistant luminal and triple-negative subpopulations at advanced stages, retesting for low HER2 expression using radiomic guidance may be an important option for recruiting patients for ongoing clinical trials of anti-HER2 therapies.

ML is a computational approach that leverages data to enhance performance and make accurate predictions. In this study, we used 6 supervised classification algorithms: LR, LDA, SVM, DT, RF, and AB. They are commonly used to classify breast lesions or differentiate between the molecular subtypes of BC.^[41]^ LR and LDA aim to identify linear relationships between input data and outcome variables, providing simple and interpretable results.^[42]^ The SVM operates by transforming data to enable the separation of classes through a hyperplane, which is represented as a line in a 2-dimensional space. Support vectors located closest to this hyperplane play a crucial role in the classification.^[43]^ The RF algorithm constructs multiple DTs with varying depths by using random subsets of data and features at each split to create uncorrelated trees. The final prediction was the average of all trees, resulting in an unbiased model.^[43]^ DT is specifically used for early BC detection and organizes the dataset into smaller subsets for classification.^[44]^ AB addresses these challenges by combining “weak” models to form a diverse and accurate model with strong generalization capabilities.^[42,45]^

To date, scholars have applied ML algorithms combined with clinical or imaging data to build predictive models to further improve the accuracy.^[24,35]^ To obtain the best prediction model, some scholars have compared the prediction performances of different ML algorithms.^[24,35,46]^ Zhang^[35]^ developed 5 ML models based on the radiomic features of dynamic contrast-enhanced MRI and non-mono-exponential model-based diffusion-weighted imaging to predict HER2+ and HER2− status, including LR, RF, AB, SVM, and LDA. Their findings revealed that both the RF and AB models exhibited superior predictive abilities compared to the other 3 models. Similarly, Sheng et al^[46]^ found that the RF model outperformed other ML models by extracting radiomic features from dynamic contrast-enhanced-MRI to predict HER2-overexpressed and non-HER2-overexpressed BC.

In contrast to previous studies, our study revealed that LDA and LR exhibit superior predictive performance. We hypothesize that this disparity may be due to the fact that previous models did not consider the HER2-low category, despite this category representing the majority of BCs and being the therapeutic target of the new antibody–drug conjugates.

### 4.1. Limitations

First, this was a prospective study with a relatively small sample size; a larger sample size is required for further studies. Second, we used an immunohistochemical surrogate as a prognostic biomarker for BC rather than whole-gene sequencing. Third, the ROI segmentation in this study was performed manually, which may introduce potential errors due to human involvement. Finally, it is unclear whether the current predictive models can be applied to different imaging protocols and machines in other institutions.

## 5. Conclusion

ML models based on CE-CBBCT radiomics features achieved excellent performance in the preoperative prediction of HER2-low BC, and LDA and LR showed superior predictive capabilities, suggesting that they could be valuable tools for guiding precise and personalized targeted therapies. These are preliminary results, and a larger sample size is required to validate our conclusions.

## Acknowledgments

All authors thank the radiologists and patients at the Guangxi Medical University Cancer Hospital. We thank Paperpal (https://preflight.paperpal.com) for the English language review of this manuscript.

## Author contributions

**Conceptualization:** Xianfei Chen.

**Data curation:** Xianfei Chen.

**Formal analysis:** Danke Su.

**Funding acquisition:** Xianfei Chen.

**Investigation:** Xianfei Chen.

**Methodology:** Xianfei Chen, Minghao Li.

**Project administration:** Danke Su.

**Resources:** Danke Su.

**Software:** Xueli Liang.

**Supervision:** Danke Su.

**Validation:** Xianfei Chen, Xueli Liang.

**Visualization:** Xianfei Chen, Minghao Li.

**Writing – original draft:** Xianfei Chen.

**Writing – review & editing:** Danke Su.

## Supplementary Material


