# Supplementary material for: Performance evaluation of ML models for preoperative prediction of HER2-low BC based on CE-CBBCT radiomic features: A prospective study
Source: Medicine (Baltimore). 2024 Jun 14;103(24):e38513. doi: 10.1097/MD.0000000000038513 (PMC11175967; doi:10.1097/MD.0000000000038513)
Supplement: Supplementary file 1 [file medi-103-e38513-s001.docx]

**Supplementary Materials**

**Machine Learning Algorithms:**

1. **Linear Discriminant Analysis(LDA):** A classifier with a linear decision boundary, generated by fitting class conditional densities to the data and using Bayes’ rule. The model fits a Gaussian density to each class, assuming that all classes share the same covariance matrix. The fitted model can also be used to reduce the dimensionality of the input by projecting it to the most discriminative directions. more details can be found at <http://scikit-learn.org/0.16/modules/generated/sklearn.lda.LDA.html>

**(2)Random Forest(RF):** A random forest is a meta-estimator that fits several decision tree classifiers on various sub-samples of the dataset and uses averaging to improve the predictive accuracy and control over-fitting. Trees in the forest use the best-split strategy, i.e. equivalent to passing splitter="best" to the underlying Decision Tree Regressor. The sub-sample size is controlled with the max_samples parameter if bootstrap=True (default), otherwise the whole dataset is used to build each tree. more details can be found at <https://scikit-learn.org/stable/modules/generated/sklearn.ensemble.RandomForestClassifier.html>

**(3)Logistic Regression(LR):** Logistic regression is a well-established technique that, despite its name, is used more generally as a classifier. Logistic regression models have a fixed number of parameters that depend on the number of input features, and they output categorical prediction. It is similar to linear regression, where several points are fitted to a line, minimizing a function like the mean squared error (MSE). Logistic regression instead fits the data to a sigmoid function from 0 to 1, and, when the output is less than 0.5, the example is assigned to a class, else it is the other. More details can be found at <http://scikit-learn.org/stable/modules/generated/sklearn.linear_model.LogisticRegression.html>

**(4)Support Vector Machine(SVM):** SVMs are based on the idea of finding a hyperplane that best divides the set of training examples into 2 classes. Support vectors are the examples nearest to the hyperplane, the points of a data set that, if removed, would alter the position of the dividing hyperplane. A hyperplane is a line that linearly separates and classifies a set of data. The goal then is to determine the formula for a plane that best separates the examples. This is called a hyperplane because the dimensionality of the plane is the dimension of the examples (and remember each example is a vector of features). It is common to remap the points from simple n-dimensional space to a different type of space if that can produce a better separation of points. There also are hyperparameters (a variable that is external to the model and whose value cannot be estimated from data) that have an impact on how a model develops. For instance, in cases of SVMs, a penalty must be assigned to an example that is on the wrong side of the decision plane. The hyperparameter is the weighting of that penalty—the weighting of no examples really wrong (therefore, assigning a high power to the error) versus fewer examples wrong, even if those are really wrong. More details can be found at https://scikit-learn.org/stable/modules/svm.html

**(5)Ada-boost(AB):** The core principle of AdaBoost is to fit a sequence of weak learners (i.e., models that are only slightly better than random guessing, such as small decision trees) on repeatedly modified versions of the data. The predictions from all of them are then combined through a weighted majority vote (or sum) to produce the final prediction. The data modifications at each so-called boosting iteration consist of applying weights $w_{1}$, $w_{2}$, …, $w_{N}$ to each of the training samples. Initially, those weights are all set, so that the first step simply trains a weak learner on the original data. For each successive iteration, the sample weights are individually modified and the learning algorithm is reapplied to the reweighted data. At a given step, those training examples that were incorrectly predicted by the boosted model induced at the previous step have their weights increased, whereas the weights are decreased for those that were predicted correctly. As iterations proceed, examples that are difficult to predict receive ever-increasing influence. Each subsequent weak learner is thereby forced to concentrate on the examples that are missed by the previous ones in the sequence. More details can be found at <http://scikit-learn.org/stable/modules/generated/sklearn.ensemble.AdaBoostClassifier.html>

https://scikit-learn.org/stable/modules/ensemble.html#adaboost

**(6)Decision tree(DT):** Decision Trees are a non-parametric supervised learning method used for classification and regression. The goal is to create a model that predicts the value of a target variable by learning simple decision rules inferred from the data features. A tree can be seen as a piecewise constant approximation. More details can be found at <https://scikit-learn.org/stable/modules/tree.html>

more information can be found on https://scikit-learn.org/stable/user_guide.html#
